# Emotion perception across cultures: the role of cognitive mechanisms

**DOI:** 10.3389/fpsyg.2013.00118

**Published:** 2013-03-12

**Authors:** Jan B. Engelmann, Marianna Pogosyan

**Affiliations:** ^1^Laboratory for Social and Neural Systems Research, Department of Economics, University of ZurichZurich, Switzerland; ^2^Division of Public Administration, International Christian UniversityTokyo, Japan

**Keywords:** emotion, culture, display rules, attention, amygdala

## Abstract

Despite consistently documented cultural differences in the perception of facial expressions of emotion, the role of culture in shaping cognitive mechanisms that are central to emotion perception has received relatively little attention in past research. We review recent developments in cross-cultural psychology that provide particular insights into the modulatory role of culture on cognitive mechanisms involved in interpretations of facial expressions of emotion through two distinct routes: display rules and cognitive styles. Investigations of emotion intensity perception have demonstrated that facial expressions with varying levels of intensity of positive affect are perceived and categorized differently across cultures. Specifically, recent findings indicating significant levels of differentiation between intensity levels of facial expressions among American participants, as well as deviations from clear categorization of high and low intensity expressions among Japanese and Russian participants, suggest that display rules shape mental representations of emotions, such as intensity levels of emotion prototypes. Furthermore, a series of recent studies using eye tracking as a proxy for overt attention during face perception have identified culture-specific cognitive styles, such as the propensity to attend to very specific features of the face. Together, these results suggest a cascade of cultural influences on cognitive mechanisms involved in interpretations of facial expressions of emotion, whereby cultures impart specific behavioral practices that shape the way individuals process information from the environment. These cultural influences lead to differences in cognitive styles due to culture-specific attentional biases and emotion prototypes, which partially account for the gradient of cultural agreements and disagreements obtained in past investigations of emotion perception.

## Introduction

Faces are of central importance for social communication. They can provide a crucial window into the mental states of other people via gaze direction, which indicates focus and shifts of attention, and expression, which can reveal emotional states. The biological significance of facial cues is underlined by converging evidence from developmental and cross-cultural psychology, as well as cognitive neuroscience. As early as 9 minutes after birth, infants show attentional preferences for faces over similar objects (Johnson et al., 1991) that develop into identity discriminatory abilities within as little as 3 months (Kelly et al., 2005, 2007). Infants also perceive different facial expressions at a very early age, as indicated by their ability to imitate facial gestures by the time they are 12 days old (Meltzoff and Moore, 1977). These findings suggest a special status of face perception (for review see McKone et al., 2009), which is thought to contribute to the development of cognitive skills, such as language and mentalizing (Meltzoff and Decety, 2003). Cognitive neuroscience investigations using human neuroimaging and monkey electrophysiology have gathered considerable evidence for the existence of neural architecture that specializes in face perception consisting of core regions involved in visual feature analysis and extended regions involved in interpreting emotional expressions (for reviews see Haxby et al., 2000, 2002; Kanwisher, 2000; Ishai, 2008). Finally, cross-cultural investigations have demonstrated above-chance recognition accuracies for basic emotions across multiple literate and illiterate cultures (e.g., Ekman et al., 1969, 1987; Ekman and Friesen, 1971; Izard, 1971; Matsumoto and Ekman, 1989). These results have been interpreted as indicating that similar patterns of facial muscle movements are produced by people around the world to express basic emotions (Ekman et al., 1969). Recent evidence partially supports this notion, particularly for happy and sad expressions (Jack et al., 2012b). Taken together, converging evidence from different fields revealing early developmental onset of skills related to face perception, neural architecture specializing in face perception and relative cultural consensus in interpretations of facial expressions of basic emotions suggests that core aspects of expression and recognition of emotion have an evolutionary and biological origin (Darwin, 1872; Tomkins, 1962; Ekman et al., 1969; Izard, 1971; Susskind et al., 2008).

While results from early cross-cultural investigations of emotion perception support the presence of some level of cultural universality in emotion perception and production (Ekman et al., 1969; Ekman and Friesen, 1971; Izard, 1971), more recent results reveal cross-cultural differences in cognition and behavior (for review see Nisbett and Masuda, 2003), including the domain of emotion perception (e.g., Ekman et al., 1987; Matsumoto and Ekman, 1989; Biehl et al., 1997; Yrizarry et al., 1998; Nisbett et al., 2001; Matsumoto et al., 2002; Jack et al., 2012b). Prior experiments employing full-face and high-intensity expressions, have identified a gradient of cultural agreement that is greatest for positive emotions, such as happiness, and lowest for negative emotions, such as fear and anger (Ekman et al., 1987; Matsumoto, 1990). In conjunction with evidence supporting the notion of cultural accents in facial expressions (Marsh et al., 2003; Elfenbein et al., 2007), these findings have sparked a debate about the relative contributions of nature versus nurture to the expression and perception of emotion. The current consensus is that innate, biological factors, such as genes and brain systems, are significantly shaped by cultural and social contexts during development. It is these complex interactions between biology and context that contribute to the observed behavioral patterns of cultural agreement and disagreement in identifying expressions of affect (e.g., McCrae et al., 2000; Adolphs, 2001; Elfenbein and Ambady, 2003).

An important question that has emerged from decades of cross-cultural psychological research pertains to the cognitive channels through which culture shapes emotion perception in a manner consistent with previously observed patterns of cultural agreement and disagreement. While this question has received relatively little attention to date (see however, Nisbett and Masuda, 2003; Park and Huang, 2010), we review recent findings that have shed light on cultural influences on cognitive mechanisms involved in extracting and categorizing emotional expressions. Specifically, we integrate two recent developments in cross-cultural psychology that provide particular insights into the modulatory role of culture on interpretations of emotional expressions and underlying cognitive mechanisms, namely (a) investigations of cross-cultural differences in emotion intensity perception that underline the impact of **display rules** on **emotion prototypes**, and (b) investigations of cross-cultural differences in feature extraction during decoding of facial expressions of emotions that underline the influence of culture on **cognitive styles.**

## Perception of emotion intensity varies across culture

Cultural variation in emotion intensity perception has been well-documented in past research (e.g., Ekman et al., 1987; Matsumoto and Ekman, 1989; Matsumoto, 1990; Biehl et al., 1997; Yrizarry et al., 1998; Matsumoto et al., 2002). A typical approach in research on emotion intensity perception has been to request category judgments and intensity ratings sequentially. In the majority of prior studies, intensity ratings were obtained via a single Likert-type scale, which asks participants about their perception of the intensity of the emotion portrayed by a poser (e.g., Matsumoto and Ekman, 1989; Matsumoto, 1993; Biehl et al., 1997). One of the most consistent cultural differences revealed in past research investigating emotion intensity perception is the tendency of Americans to rate the same expressions more intensely compared to Japanese participants across a range of emotions including happiness, sadness, and surprise (Ekman et al., 1987; Matsumoto and Ekman, 1989; Matsumoto, 1990). These differences in intensity ratings are independent of race or gender of the poser (Matsumoto and Ekman, 1989; Matsumoto, 1990) and have even been observed among ethnic groups within one culture (Matsumoto, 1993). In an effort to obtain a more inclusive picture of cultural effects on emotion perception, one investigation analyzed data obtained from multiple intensity rating scales covering all basic emotions for a given expression, including target scales (e.g., happiness ratings when happiness was displayed) and non-target scales (e.g., surprise ratings when happiness was displayed; Yrizarry et al., 1998). Results revealed complex cultural differences in intensity ratings on multiple emotion scales, including non-target emotions.

One shortcoming of earlier investigations of emotion intensity perception is the use of a single intensity rating scale. This type of scale can introduce ambiguity about the nature of the task, which participants could interpret as requesting intensity ratings of the external display of affect, or the subjective experience of the poser (Matsumoto, 1999). Matsumoto (1999) addressed this shortcoming by employing two separate scales, one assessing external display and one assessing intensity of emotion. Results indicated that American and Japanese participants distinguish between external displays and internal experiences of affect, but differentially so. Specifically, Americans gave higher ratings than Japanese participants to external appearance, while the Japanese rated internal feelings of posers significantly higher than Americans. In a follow-up experiment, Matsumoto et al. (2002) created expressions of intermediate and exaggerated intensities by interpolating (morphing) emotional expressions with neutral expressions of the same individual. In this case, American participants rated external displays significantly higher than internal experiences when viewing high intensity expressions. However, when viewing low intensity expressions, this difference was no longer found. Japanese participants, on the other hand, rated low intensity expressions as significantly higher in internal experience relative to external display, but no difference was found for high intensity expressions.

These results demonstrate a discrepancy between the percept of emotional expressions and inferences about internal states of posers. Importantly, this discrepancy is modulated by culture in a manner consistent with predictions made on the basis of display rules (e.g., Ekman et al., 1969; Matsumoto, 1990; Matsumoto et al., 2002, 2008). Display rules can be described as culturally-specific normative prescriptions about the appropriateness of the presence and intensity-level of emotional expressions in different social settings, that is when, how and to whom emotions ought (not) to be displayed (Matsumoto et al., 2008). Procedures for the management of emotion displays are learned during development within a particular culture and include amplification and deamplification, as well as qualification, masking, and neutralization (e.g., Ekman et al., 1969; Matsumoto et al., 2008). A classical study investigating facial expressions in a stressful situation across cultures is often cited as evidence for the existence of display rules (Ekman, 1971). In this study, American and Japanese participants viewed stressful films while their facial expressions were recorded in two conditions, when alone, or in the presence of an experimenter. In the alone condition, participants from both cultures produced similar facial expressions of basic emotions during the viewing of the film. When an experimenter was present in the room, Japanese participants displayed a greater propensity to mask their negative emotions through smiling compared to American participants, who tended to continue to express their negative emotions despite the presence of the experimenter. The experimenters argued that these cultural differences in displays of affect occurred due to the Japanese display rule of concealing negative affect in social settings (Ekman, 1971). More recent research confirms these early findings. Specifically, it has been shown that Western, individualistic cultures tend to endorse emotion expression, while Asian, collectivistic cultures encourage the control of expressions of affect to maintain group harmony (Markus and Kitayama, 1991; Heine et al., 1999; Matsumoto et al., 2005, 2008). Thus, the role of display rules in regulating expressions of emotion to maintain their appropriateness in a variety of contexts has been well-documented.

Taken together, although relatively few studies have addressed the issue of emotion intensity perception, past research has provided consistent evidence for the presence of cultural differences in interpreting facial expressions of emotion. Among the notable shortcomings of prior studies is the use of a single rating scale and unnatural stimuli, either in the form of caricature facial expressions of emotion (Russell et al., 2003; Barrett et al., 2007; Scherer et al., 2011), or potentially artifactual stimuli created through morphing emotional expressions with neutral expressions (Calder et al., 1996). Of note, while the findings of the investigations reviewed above have made an important contribution in outlining cultural differences in emotion intensity perception, the methodologies employed so far do not allow for investigating specific cognitive mechanisms that contribute to observed differences. More recent experiments have made progress in identifying underlying cognitive mechanisms that contribute to cultural differences in emotion perception (e.g., Jack et al., 2009, 2012a,b; Pogosyan and Engelmann, 2011).

## Cognitive mechanisms 1: culture modulates emotion perception by shaping mental representations

Context has a significant influence on multiple perceptual, cognitive, affective and related neural mechanisms that impact judgment and decision-making (Engelmann and Hein, 2013). Context is also crucial for interpretations of emotional expressions (for review see Barrett et al., 2011). One important factor that can have modulatory effects on how context is interpreted is culture. In a recent study, Masuda et al. (2008) revealed that when inferring emotions of other people, the Japanese tend to rely more heavily on social context than the Americans, who relied solely on the target person rather than the people in the group surrounding her/him. Despite the importance of context for emotion judgments, the majority of investigations of emotion perception have been carried out in a contextual vacuum. In an effort to address this limitation, a recent investigation embedded emotion intensity perception within a context that “naturally” occurs across cultures, namely advertising (Pogosyan and Engelmann, 2011). Specifically, beauty advertisements were selected as an ecologically valid stimulus set because they offer various advantages over the standard stimulus sets typically used in investigations of emotion intensity perception, including (1) their worldwide prevalence, thereby providing a naturalistic backdrop for investigations of emotion perception, (2) a rigorous selection process before going to print, thereby ensuring the authenticity of portrayed emotions, and (3) relatively homogeneous levels of attractiveness, thereby controlling for potential attractiveness confounds.

American, Russian, and Japanese female participants rated emotional expressions of fictitious beauty advertisements on various intensity domains, using multiple emotion adjectives as response alternatives including high-arousal (excited, elated, enthusiastic), positive (happy, content, satisfied) and low arousal (calm, relaxed, peaceful) items. These simultaneously collected intensity ratings provided a finely grained glimpse into response patterns that enabled investigations of the degree of categorization of facial expressions into distinct arousal categories across culture via categorical difference scores. Furthermore, each model was shown twice, once portraying high intensity positive emotions and once, low intensity positive emotions, in order to investigate cultural differences in the perception of emotion displays of varying intensity via perceptual difference scores.

**Figure 1:**
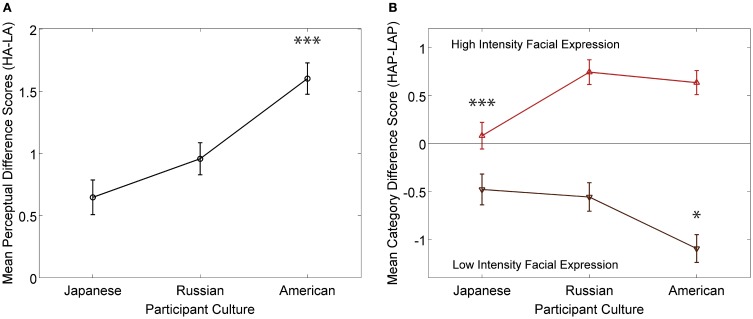
**Summary of main results from Pogosyan and Engelmann (2011).** Results show cultural differences in the underlying cognitive mechanisms involved in emotion intensity perception. **(A)** Cultural differences in distinguishing between high and low intensity expressions of affect were observed, such that American participants differentiated between intensity to a greater degree than other cultures. **(B)** Cultural differences in the way high and low intensity expressions of positive affect are categorized across culture. Japanese participants did not classify high intensity expressions as either high or low arousal, while American participants identified low intensity expressions as significantly lower arousal compared to other cultures. ^***^p < 0.005; ^**^p < 0.01; ^*^p < 0.05.

The findings from Pogosyan and Engelmann (2011) revealed cross-cultural agreement, as well as variation in the perception and categorization of facial expressions. While findings indicated no significant rating differences across cultures for either low or high intensity facial expressions on response alternatives reflective of intermediate arousal percepts (e.g., happy), cross-cultural differences for high and low arousal response alternatives were revealed. Specifically, American participants perceived low intensity facial expressions as significantly less excited than either Japanese or Russian participants, while Japanese participants perceived high intensity facial expressions as significantly calmer compared to both Russian and American participants. Based on the postulation that participants differed not only in the way they perceived emotion intensities, but also the way they followed to categorize them, separate difference scores were obtained to reveal the effect of culture on perceptual and cognitive mechanisms underlying emotion judgments. Both the perceptual and categorization difference scores provided interesting insights into the nature of the differences of the perceptual and evaluatory mechanisms across cultures. Perceptual difference scores revealed that, while all cultures differentiated between high and low intensity facial expressions, American participants did so to a significantly greater degree (Figure 1A). Such high degree of differentiation between high and low intensity displays of positive affect among American participants is indicative of higher intensity prototypes compared to Japanese and Russian participants. Category difference scores revealed that American participants distinctly categorized displays of positive affect into low and high intensity categories. Japanese and Russian participants, on the other hand, deviated significantly from clear categorization. Specifically, high intensity expressions were judged to be equal in high and low arousal categories by the Japanese (Figure 1B), and to be equal in high and intermediate arousal categories by Russian participants. Together, these results point to cultural variations in specific cognitive mechanisms, namely the differentiation between displays of affect intensities and the categorization of expressions of positive affect.

These results inform underlying mechanisms through which culture-specific display rules that govern the production of emotional expressions impact emotion perception. Display rules result in sustained exposure to culture-specific intensity levels of facial expressions. This leads to culture-specific learning about facial expressions during critical developmental stages and throughout the lifespan, thereby shaping mental representations of facial expressions of affect. Mental representations can be thought of as prototype emotional expressions that are used when meaning is assigned to percepts. Given the dominance of particular display rules within a given culture (Matsumoto et al., 2005, 2008), culture-specific hypotheses about their impact on shaping prototype emotion, and consequently emotion perception, can be generated: (1) cultures that endorse emotion displays would be expected to have greater mean intensity and standard deviation levels of endorsed emotions; (2) cultures that prescribe de-amplification of specific emotions, on the other hand, would be expected to have lower intensity prototypes and lower standard deviations of emotional expressions that are regulated by display rules. Results from Pogosyan and Engelmann (2011) support this notion. The clear-cut differentiation between high and low intensity expressions of positive affect is reflective of high intensity prototype emotions among American participants. The lack of categorization of high intensity expressions into distinct arousal domains by Japanese and Russian participants is reflective of blended emotion prototypes that fall between specific intensity domains. These results agree with predictions made on the basis of display rules specific to the cultures that were investigated (Matsumoto et al., 2008).

## Cognitive mechanisms 2: culture modulates emotion perception by shaping attentional biases

Display rules likely influence and interact with culturally variable cognitive processing styles. For instance, Western cultures have been shown to adopt feature processing strategies, while Asian cultures demonstrate a disposition to employ holistic strategies (e.g., Nisbett and Masuda, 2003). This notion is supported by considerable evidence demonstrating cultural differences in relative versus absolute size judgments (Kitayama et al., 2003), categorical reasoning (Norenzayan et al., 2002), perceptual processing strategies (Damjanovic et al., 2010), as well as attentional mechanisms, such as change blindness sensitivity (Nisbett and Masuda, 2003; Masuda and Nisbett, 2006) and eye movement patterns (Chua, 2005; Blais et al., 2008; Masuda et al., 2008; Jack et al., 2009). Jointly, these results identify cultural differences in cognitive processing styles that underline the wide-ranging and general effects of culture on cognition. Recent experiments have made important progress in shedding light on cultural differences in cognitive processing strategies involved in emotion perception and categorization (Blais et al., 2008; Jack et al., 2009, 2012a). Specifically, results from a series of experiments suggest that participants from different cultures sample information differently from faces during face identification (Blais et al., 2008) and categorization of facial expressions of affect (Jack et al., 2009, 2012a,b). Using eye tracking as a proxy for overt attention to face regions demonstrated a systematic bias to attend to a more limited set of face regions in East Asian participants relative to Western participants. During face identification, East Asian participants were shown to focus on a central region around the nose, while Western participants sampled more broadly from the eyes and mouth (Blais et al., 2008). During emotion categorization, a systematic bias to attend to eye regions was related to a significant deficit in categorizing fearful and disgusted facial expressions in East Asian participants (Jack et al., 2009). Western participants, on the other hand, had a propensity to sample from both eye and mouth regions and made relatively less categorization mistakes.

A more recent investigation replicated these findings using reverse correlation for reconstructing internal representations from average white noise templates that biased judgments (Jack et al., 2012a). This method yielded culture-specific templates for each of the basic emotions that revealed significant differences in the features that are predominantly used by East Asian compared to Western participants during face perception. One intriguing finding that provides a putative explanation for the attentional bias to the eye region exhibited by East Asian participants, is that gaze shifts seem to be considered components of facial expressions of affect among East Asian participants (Jack et al., 2012a). Interestingly, the importance of the eye region for inferring emotional states in Asian cultures is underlined by differences between emoticons used in Asian and Western cultures: emotions are mostly communicated via symbols that vary eye shape in Asian cultures and mouth-shape in Western cultures. Further evidence for culture-specific attentional biases is provided by a recent investigation that employed facial expressions of emotion in which eye and mouth regions were independently manipulated (Yuki et al., 2007). Yuki et al. (2007) demonstrated that faces displaying conflicting expressions, such as sad eyes combined with a happy mouth, are perceived differently by Japanese and American participants. When inferring emotions, Japanese participants weighted the eye region more strongly, while American participants were influenced relatively more strongly by the mouth region. Together, these results underline the presence of culture-specific top–down attentional mechanisms responsible for extracting information for emotion categorization from faces. An intriguing neural hypothesis is that these attentional patterns are in part guided by the **amygdala**, which is considered to be crucially involved in selective information processing of biologically significant stimuli (Pessoa, 2010). Recent evidence from cultural neuroscience, demonstrating consistent cultural differences in amygdala activation patterns during face perception (Moriguchi et al., 2005; Chiao et al., 2008; Adams et al., 2010; Derntl et al., 2012, 2009), underlines this notion.

## A cascade of cultural influences on cognition

**Figure 2:**
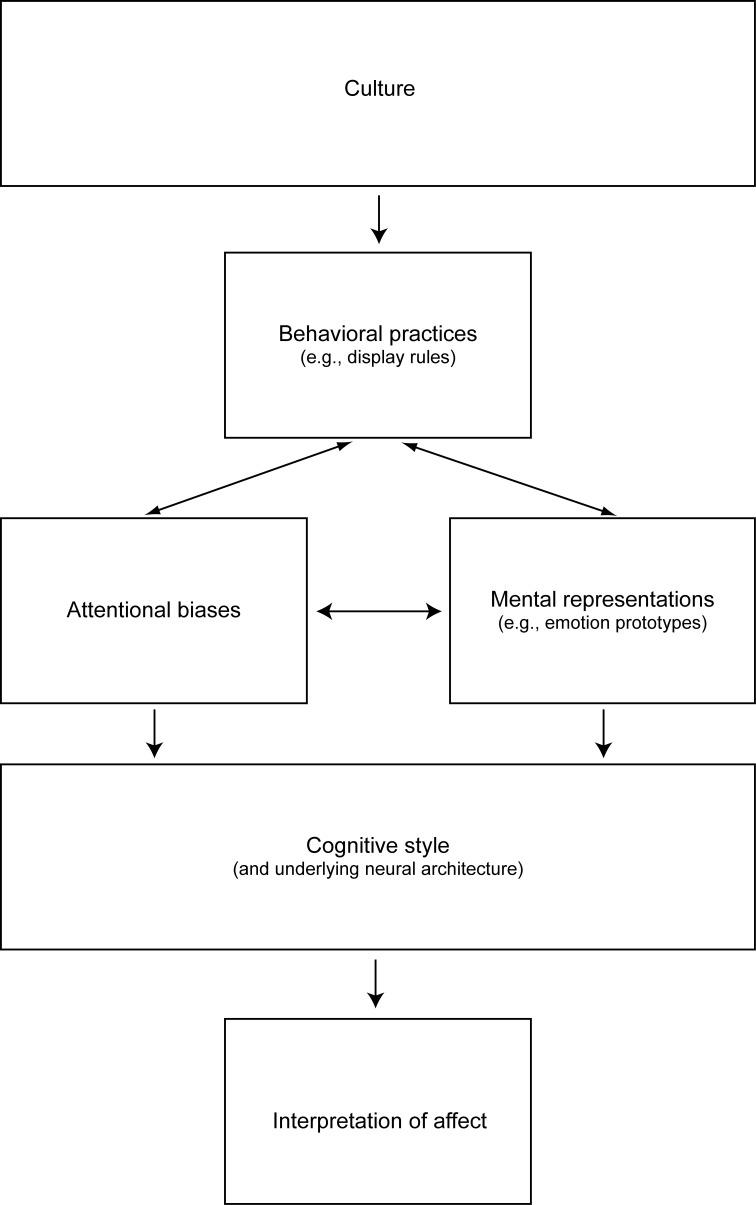
**Cascade of cultural influences on interpretations of emotional expressions.** Culture shapes display rules and behavioral practices, which, through learning, influence specific cognitive mechanisms, such as attentional biases and mental representations. Specifically, sustained exposure to cultural practices influences the way individuals process information from the environment. For example, display rules may lead to culture-specific enhancements and reductions in exposure frequency and intensity of specific emotion displays. Display rules thereby mediate perceptual learning about facial expressions, which in turn shapes attentional biases and mental representations. Sustained exposure to such behavioral practices leads to the formation of cognitive styles, which shape how emotional expressions are interpreted across cultures. Culture-specific cognitive styles are mediated by underlying neural mechanisms, which have been the focus of investigations in cultural neuroscience.

The results reviewed above demonstrate wide-ranging influences of culture on cognitive mechanisms involved in emotion perception, including mental representations and, relatedly, emotion prototypes, as well as attentional biases. One question that arises is how these different processes interact with one another. Figure 2 shows a possible cascade of cultural influences on cognitive mechanisms involved in interpretations of facial expressions of emotion. At the highest level of the proposed hierarchy is culture, which imparts the regulatory norms and pertinent social platforms that provide idiosyncratic meaning and guidelines for emotional behavior (e.g., Tomkins, 1962). Considerable inter-disciplinary evidence suggests that cultural context shapes perceptual experiences, which, in turn, modulate cognitive mechanisms and related neural systems (for review see Park and Huang, 2010).

Display rules comprise one important aspect of culture-specific behavioral practices, which mediate perceptual learning about facial expressions via enhancements and reductions in exposure levels to specific emotion displays. This notion is supported by a recent computational modeling study in which the effect of display rules on learning was simulated via manipulating exposure frequency to particular emotional expressions during training of a biologically plausible neural network model (Dailey et al., 2010). Results from this study demonstrate that the best match between model and Japanese participants' performance was achieved when training the computational model with a face set that significantly subsampled angry expressions. This finding has both general implications, in that it underlines the importance of culture-specific learning in interpreting facial gestures, as well as specific implications, in that it supports the presence of display rules that discourage the expression of negative emotions in Japan, as reported elsewhere (Safdar et al., 2009). Finally, culture-specific learning can have a significant impact on brain systems implicated in perceptual decision-making. Recent results in cognitive neuroscience provide evidence that sustained exposure to particular behavioral practices can change not only cognitive processes related to category organization (Polk and Farah, 1998), but also structure and function of relevant neural systems (e.g., Maguire et al., 2000; Gaser and Schlaug, 2003; Draganski et al., 2004; Boyke et al., 2008). We therefore propose that cultural frameworks providing specific belief-, value-, and knowledge-sets, as well as behavioral practices, can shape cognitive and neural mechanisms related to interpretations of facial expressions (e.g., Park and Huang, 2010).

The intermediate level of the cascade is represented by cognitive style, which is in part determined by attentional biases and mental representations. Cognitive style reflects how individuals habitually extract and process information from the environment. Results reviewed above have demonstrated important ways in which culture shapes cognitive style, namely by imparting attentional biases during face perception (Blais et al., 2008; Jack et al., 2009, 2012a,b). Given our knowledge of the functional neuroanatomy of attention (e.g., Ungerleider, 2000; Engelmann et al., 2009), we hypothesize that such culturally transmitted attentional biases can be revealed in brain regions within the fronto-parietal attention network known to be involved on top–down attentional control, such as the frontal eye fields, ventral premotor cortex, superior parietal lobule, and intraparietal sulcus. We are hopeful that future research in cultural neuroscience will identify the neurobiological mechanisms responsible for culture-specific attentional biases evident during face perception. Cultural influences on information processing also impact mental representations (Jack et al., 2012a), such as emotion prototypes. Findings from a recent investigation on emotion intensity perception reviewed above are consistent with the notion that significant cultural differences exist in the intensity levels of positive emotion prototypes (Pogosyan and Engelmann, 2011). Together, culture-specific cognitive styles can account for some of the cultural differences in emotion perception commonly observed in past research.

It has to be noted that the proposed cascade of cultural influences reflects a simplified model that only considers two specific cognitive processes, namely attentional biases and mental representations. As demonstrated previously, the modulatory role of culture also extends to other cognitive mechanisms that include mathematical reasoning (Tang et al., 2006), musical processing (Nan et al., 2006, 2009), and self-representation (Zhu et al., 2007). The effects of culture on cognition may also operate by shaping linguistic environments (e.g., Lindquist et al., 2006; Roberson et al., 2007; Damjanovic et al., 2010), and decoding rules that affect interpretations of facial expressions (Matsumoto and Ekman, 1989).

## Conclusions

We have summarized findings from past research that have made important contributions to understanding how underlying cognitive mechanisms relevant to interpretations of emotion expressions are shaped by culture. Specifically, a recent investigation has demonstrated that facial expressions of differing intensity levels are perceived and categorized differently across three cultures (Pogosyan and Engelmann, 2011). Results indicate the greatest level of differentiation between intensity levels of facial expressions of emotion among American participants compared to Japanese and Russian participants. Furthermore, deviations from clear categorization of high and low intensity expressions were observed among Japanese and Russian participants. These findings suggest that culture-specific display rules shape mental representations related to the intensity of emotion displays, such as intensity levels of emotion prototypes.

Display rules and behavioral practices also shape attentional mechanisms, such as the propensity to attend to very specific features of the face when inferring identity (Blais et al., 2008) and expression (Jack et al., 2009, 2012a). One intriguing idea about how attentional mechanisms are modulated by culture is derived from the potential effects of culture-specific behavioral practices. For example, in Asian cultures, direct eye contact is often considered impolite, especially when interacting with individuals of higher status. Such practices can reduce time for sampling from multiple areas of the face. Given that the importance to infer mental states of interlocutors is consistent across cultures, individuals in Asian cultures may develop heuristics that allow the quick sampling of facial gestures. A likely scenario that is consistent with the results reviewed above is that glances are targeted at the eyes, but would be very brief to adhere to politeness norms. It is easily imagined how such culturally transmitted heuristics can influence attentional biases. Future research is needed to identify relationships between culture-specific behavioral practices and cognitive, as well as neural mechanisms. The cascade of cultural influences on cognition in Figure 2 provides a parsimonious model that can be employed to guide hypotheses for future research. The nascent field of cultural neuroscience has much promise in contributing to our understanding of how culture shapes cognitive and underlying neural mechanisms involved in face perception (for review see Han et al., 2013).

### Conflict of interest statement

The authors declare that the research was conducted in the absence of any commercial or financial relationships that could be construed as a potential conflict of interest.

## Key Concept

Display rules: Display rules are culture-specific normative prescriptions about the appropriateness of expressions of affect in different contexts. They inform individuals about when, how and to whom emotions ought, or ought not to be displayed. Emotion displays can be managed via (de-)amplification, qualification, masking, and neutralization.
Emotion prototypes: Emotion prototypes are abstract representations of emotions that comprise the most typical set of features that are shared by most instances of a particular emotion, including its expression.
Cognitive style: Cognitive style refers to individuals' dispositions in employing specific approaches to gathering and processing information from the environment. Cognitive style is reflective of habitual strategies and heuristics employed during problem solving and judgment.
Amygdala: The Amygdala is an almond shaped and complex cluster of nuclei located bilaterally in medial temporal cortex. Its functions are wide-ranging and are important for emotions, detecting biological significance and selective information processing. It is considered part of an extended network of structures that are important for face perception.
